# Synthetic Protein Circuits and Devices Based on Reversible Protein-Protein Interactions: An Overview

**DOI:** 10.3390/life11111171

**Published:** 2021-11-03

**Authors:** Stefano Rosa, Chiara Bertaso, Paolo Pesaresi, Simona Masiero, Andrea Tagliani

**Affiliations:** Department of Biosciences, Università Degli Studi di Milano, 20133 Milan, Italy; stefano.rosa@unimi.it (S.R.); chiara.bertaso@studenti.unimi.it (C.B.); paolo.pesaresi@unimi.it (P.P.)

**Keywords:** synthetic biology, protein circuits, protein-protein interactions, combinatorial libraries, peptides

## Abstract

Protein-protein interactions (PPIs) contribute to regulate many aspects of cell physiology and metabolism. Protein domains involved in PPIs are important building blocks for engineering genetic circuits through synthetic biology. These domains can be obtained from known proteins and rationally engineered to produce orthogonal scaffolds, or computationally designed de novo thanks to recent advances in structural biology and molecular dynamics prediction. Such circuits based on PPIs (or protein circuits) appear of particular interest, as they can directly affect transcriptional outputs, as well as induce behavioral/adaptational changes in cell metabolism, without the need for further protein synthesis. This last example was highlighted in recent works to enable the production of fast-responding circuits which can be exploited for biosensing and diagnostics. Notably, PPIs can also be engineered to develop new drugs able to bind specific intra- and extra-cellular targets. In this review, we summarize recent findings in the field of protein circuit design, with particular focus on the use of peptides as scaffolds to engineer these circuits.

## 1. Introduction

Living organisms, both unicellular and multicellular, respond to exogenous and endogenous signals that trigger appropriate responses, within individual cells, by using a complex system of receptors and signal-transducing proteins [1,2]. Signal transduction cascades modulate downstream cellular processes, giving rise to dynamic gene expression patterns. Transcriptional modulation can occur within minutes or hours, and involves de novo protein synthesis; for instance, hypoxia response in human cells, innate immunity in plants, and galactose sensing in yeast, are processes modulated via transcriptional reprogramming [3,4,5]. Non-transcriptional responses, on the other hand, can occur within seconds and rely on post-translational modifications and without the need for transcriptional induction and de novo protein synthesis [6,7].

In the last decade, synthetic biology has emerged as a promising field with possible applications in many aspects of human life [8]. Its applications range from bio-sensing [9], diagnostic [10], drug discovery [11] and production of living biomaterials [12]. In this context, synthetic genetic circuits offer infinite possibilities to build orthogonal and complex artificial systems designed to respond to endogenous and exogenous stimuli, similar to canonical biological signal transduction pathways. Moreover, standardization of biological parts (Bio-Bricks), together with DNA synthesis technologies, allows fast characterization of synthetic biological devices in a continuous design–build–test–learn cycle.

So far, many synthetic genetic circuits have relied on activation of target gene expression under desired circumstances [13]. Feedback loops [14], oscillators [15] and ON/OFF switches [16] have been designed to convert a desired signal in a corresponding action or behavior through the transcription/translation machinery. These strategies can be applied in several contexts however, the time required to induce transcription and translation of signal-transducing proteins, followed by further signal transduction is considerable [17].

**Figure 1:**
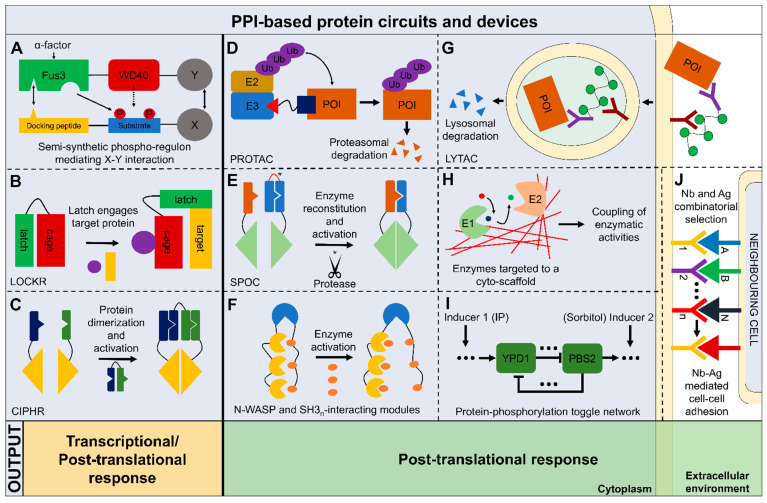
Schematic overview of the main protein-protein interaction (PPI)-based synthetic biology tools and circuits described in this review. The circuits shown here are divided in two classes based on their outputs: transcriptional/post-translational, those which have been exploited to produce both a transcriptional and post-translational output; post-translational only, those relying only on PPIs to give rise to the desired response which is directly translated to a cell behavioral change. (**A**) A semi-synthetic phospho-regulon generated with a docking peptide and a substrate peptide which are designed to dock and to be phosphorylated by Fus3, respectively, upon Fus3 activation mediated by α-factor administration. Once phosphorylated, the substrate peptide can interact with the Fus3 fused module (WD40). Fus3-WD40 chimera and phospho-regulon are linked to proteins of interest (POIs) (X and Y) which are exploited to produce different outputs [36]. (**B**) LOCKR (latching orthogonal cage/key proteins) are constituted by a cage trapping a latch, which could be displaced by a key (purple circle), leaving the latch free to engage in interactions with the desired target protein. The interaction with the target enables different kinds of output to be produced, depending on the target and the motif encoded on the latch [48]. (**C**) CIPHR (cooperatively inducible protein heterodimer) relies on de novo designed CC heterodimers which could be used as logic gates enabling different cellular functions to be performed in a programmable manner [47]. (**D**) Proteolysis-targeting chimera (PROTAC) enables the proteasomal degradation of target proteins using a small molecule (like a peptide) which function as a link between E3 ubiquitin ligase and the POI. The proximity between these two proteins enables POI ubiquitination and redirection to the proteasome [52]. (**E**) Split-protease-cleavable-orthogonal-CC-based (SPOC) implements de novo CC design to reconstitute the activity of split proteases after the cleavage (*) and displacement of an autoinhibitory domain [46]. (**F**) Ultrasensitive protein switch based on the N-WASP output domain (blue), retaining a well-defined catalytic activity, and combined with a different number of SH3 (yellow) interaction modules. Once SH3-binding peptide (orange) is administered exogenously, N-WASP is activated. It was shown that increasing the number of SH3 interaction modules increases ultra-sensitivity [39]. (**G**) Lysosomal-targeting chimeras (LYTACs) enables lysosome-mediated degradation of extracellular and membrane POIs. Using a lycopolypeptide ligand (sinusoid line with green circles) conjugated to an antibody (purple), a POI could be internalized by engaging lysosome-targeting receptor (dark red) [57]. (**H**) De novo designed CC cyto-scaffold encompassing entirely the *E. coli* cytoplasm (red stripes) were used to couple enzymatic activities of E1 (Enzyme 1, pyruvate decarboxylase) and E2 (Enzyme 2, alcohol dehydrogenase) to physically link their metabolic activity (ethanol production) thanks to their co-localization [51]. (**I**) A bistable toggle switch based on reversible (phosphorylation-dependent) PPIs. The network comprises positive feedback regulation which can repress each other through two branches, and the possibility to switch between two states of the system using two different inputs [isopentenyl adenine (IP) and sorbitol]. This phosphorylation toggle responds in seconds, displays long-term bistability, and retains ultra-sensitivity. It was used to control the inhibition of yeast cell budding by redirecting a cytoskeletal protein to the nucleus [6]. (**J**) Synthetic platform enabling multi-cellular self-assembly of *E. coli* cells. Combinatorial libraries encoding for nanobodies (Nb) and antigens (Ag) exposed on the bacterial outer membrane enabled the selection of specific Nb-Ag interactions to control the construction of well-defined multicellular patterns and morphologies [62].

A speedier method, which allows cells to relay an external stimulus, and directly elicits a cellular response, is the employment of reversible protein-protein interactions (PPIs), thus bypassing the need for transcriptional induction and translation of the signal transducer, which can be already expressed in the cell a priori and activated only under desired circumstances. PPIs can directly trigger a cellular response by sequestration of enzymes, degradation of target proteins or other mechanisms [18]. PPIs rely on mutual recognition of interacting domains in the crowded environment of the cell. These interactions are usually masked and can be promoted only under certain circumstances, e.g., binding of allosteric regulators, conformational changes of upstream regulators, post-translational modifications. Recently, it has been shown by Mishra et al., 2021 [6], that a synthetic protein circuit, based solely on reversible PPIs, can respond within seconds and with ultra-sensitivity and can give a direct cell response, with no need of further downstream transcriptional activation; the authors developed a phosphorylation-dependent toggle switch, activated by two different signals, in which the two branches of the circuit repress each other in a feedback loop regulation. By means of reversible PPIs and multi-level regulation, this synthetic network was able to directly regulate yeast budding by different extents (Figure 1I).

Genetic circuits with transcriptional control as final outputs are constructed and tested using DNA pieces stacked together. Synthetic genetic circuits based on PPIs used to modulate cellular responses (e.g., apoptosis, metabolic adjustment) are less developed because the information and rules for PPIs are still poorly understood, especially when compared to technologies that allow the manipulation of the genetic code. For these reasons, the first PPI circuits used to build new biological tools and devices were devised to work with known proteins, as shown by the well-known example of the development of the yeast-two hybrid assay [19].

Today, computational methods, and the increasing importance of structural data, allow the design of fully synthetic and orthogonal PPI-scaffolds and linkers which can be employed for protein circuits design [20,21]. A wide range of nature-inspired (natural PPIs) or fully synthetic scaffolds have been developed so far. Moreover, these interactions can be engineered and finely tuned by small molecules or physical stimuli. Notably, synthetic PPIs can be exploited also in drug discovery, by targeting endogenous proteins and inducing new behaviors under desired circumstances [22].

In this review we explore new and old aspects of protein circuits design, encompassing novel and/or established strategies to develop fast-responding, orthogonal and non-orthogonal synthetic circuits and devices based solely on PPIs. These systems can be developed both to drive a new transcriptional program in the cell or to directly give an adaptive response to the environment. Moreover, recent advances in the field of structural biology and molecular modeling are also discussed, as these can greatly improve the development of this field of synthetic biology. Emphasis will be laid also on the use of combinatorial libraries to isolate new scaffolds or drugs, and on the use of small peptides as convenient mediator of PPIs. The synthetic circuits discussed in this review are summarized in Figure 1.

## 2. Protein Modules for Protein-Protein Interaction (PPI)-Based Synthetic Circuit Design

PPIs are a cornerstone of every single biological process, such as signal transduction, differentiation, and many others. Protein interaction motifs and binding regions are extremely versatile as building blocks to construct synthetic cellular networks [23], and the use of canonical (natural) or artificial PPI domains in synthetic pathways might allow for switching the cell behavior at will.

The building blocks for engineering PPIs can be determined by studying protein complexes. Dove and Hochschild (1988) were pioneers in this field; they took advantage of the interaction between the dimerization domain of the yeast Gal4 transcriptional activator and the Gal11 protein, a subunit of the RNA Polymerase II holoenzyme (indispensable to activate the transcription), to guide association between the α-subunit of *E. coli* RNA polymerase and DNA-binding proteins, and trigger transcription [24]. Another example is the modification of an existing phage λ circuit, where the phage λ Cl oligomerization region has been fused to different DNA-binding domains, forming new transcription regulators with increased digital-like (ON/OFF transition) behavior compared to the pre-existing phage system [25,26].

The engineered PPI networks can be time-modulated; thus, the desired response can be induced only when required. Metabolite-responsive transcriptional regulators have been created by fusing zinc finger DNA-binding domains to a maltose binding protein to produce a maltose-regulated gene expression circuit [27]. Also, the protein pair FK506 binding protein–FKB-rapamycin binding domain has been used to produce synthetic rapamycin-inducible systems [28]. In this latter case, a key role in the binding specificity of these motifs is played by a specific serine residue; moreover, the phosphorylation of this serine inhibits binding affinity [29] thus offering further regulation.

The use of natural-sourced protein domains in circuits based on PPIs can constitute a problem in developing PPI scaffolds that are orthogonal to the host, due to the widespread conservation of some of those interacting modules. For example, it has been observed in a synthetic circuit exploiting the anti-σ factors, which disrupt RNA polymerase-DNA interaction by binding to RNA polymerase σ subunit. In that case, the anti-σ factors used in the protein circuit tends to bind to the native σ factors and induced toxicity in *E. coli*. To reach orthogonality, Rhodius and colleagues (2013) isolated anti-σ factors which are orthogonal and exploitable in their synthetic platform, by testing a library of these modules toward a complementary library of RNA polymerase σ subunit [30].

To avoid non-orthogonality, PPI-based circuit design can take advantage of motifs already known for driving PPIs, which are then engineered to avoid any off-target effects. These motifs are coiled coils, leucine zippers and zinc-fingers, present in many transcription factors.

α-Helical coiled coils (CCs) consist of repetitions of seven-residue motifs (heptads) that, depending on their composition, can bind to specific structures. Since the sequence-to-structure relationship for α-helical coiled coils has been elucidated, it is relatively convenient to produce motifs able to bear PPIs with target proteins. CCs have been already employed in some synthetic circuits for transcriptional regulation in *E. coli* [31].

Cys2His2 zinc-fingers (C2H2 ZFs) mediate both protein-DNA and PPIs. C2H2 ZFs are an interesting tool for the design of synthetic proteins with novel binding specificities. The specificity of these domains depends on the types of amino acid in the first positions of the initial α-helix. Any change in such amino acids affects target specificity [33]. Such domains have been used for the construction of artificial transcription factors, and for the development of synthetic networks regulating gene expression [32]. The shuffling of the dimerization zinc finger (DNZ) domain and C2H2 ZFs can produce chimeric domains with novel protein-protein interaction specificities [33].

Also, leucine-zipper motifs can be used to trigger novel PPIs and control the assembly of protein complexes to produce a synthetic network. They have been exploited, for example, in the creation of biosensors [34], and in substitution of natural PPIs in engineered pathways in *E. coli* [31].

Despite the wide abundance of PPI modules which can be isolated and engineered, most of the previous scaffolds still rely on natural or natural-derived protein domains, which cannot always guarantee full orthogonality. Moreover, all the previous examples are based on the characterization of one or a few variants of the chosen interacting modules, which shorten the possibilities for pathway engineering. In the next section we will highlight different works about the use of peptides as fully synthetic PPI modules and their isolation through combinatorial screenings.

## 3. Peptides as Powerful Synthetic Modules for Protein Circuit Design

PPIs (or protein)-based synthetic circuits display several advantages compared to classical genetic circuits, even though their applicability is hampered by their difficult modulation [18]. To build up such protein circuits, engineered proteins needs to (i) engage interactions with other synthetic and/or endogenous proteins to transmit signals, (ii) to form assemblies, (iii) recognize specific signals and/or motifs orthogonally, (iv) specifically modify or degrade interaction partners. Moreover, these events shall be directly translated to a cellular response avoiding the need for the transcription and translation machinery.

Peptides are powerful tools to design synthetic PPI networks due to their size and properties, ease of design, flexibility and the possibility of isolating thousands of them from combinatorial libraries [35]. Peptides can be used as linkers (tags) directly fused to a protein of interest (POI) to mediate the interaction between protein pairs and/or target endogenous proteins, or they can be used as free scaffolds mediating co-recruitment of proteins under desired conditions.

Peptides were employed as modular tags to transduce signals in synthetic phosphorylation circuits in yeast cells activated by the administration of the α-factor peptide (yeast mating pheromone) (Figure 1A) [36]. A 49-residue tag, derived from proteins involved in the MAPK pathway, enabled the interaction with an endogenous scaffold upon tag phosphorylation. The interaction between two proteins—mediated by their fusion to this semi-synthetic peptide—was used to produce different outputs, like recruitment to specific cellular location, protein degradation, transcriptional responses, and feedback loops. A similar approach was also used in other works [37,38].

Alternatively, peptides have been used to produce an ultrasensitive protein switch based on the autoinhibition mediated by the cooperative binding to the neural Wiskott-Aldrich syndrome protein (N-WASP). N-WASP output domain was linked to Src Homology 3 (SH3) domains and to their peptide ligand. The sensitivity of such a system can be modulated varying the number of SH3 interaction modules (Figure 1F) [39].

Peptides can be rationally engineered to drive complex reactions inside cells and exploited to process complex signals. For instance, they could be used as biosensors for the detection of specific proteins, like in the case of the neurodegenerative-related protein Tau [40]. By mimicking a nucleic acid displacement reaction, the complementary coiled coils of the Tau inhibitor motif have been used to construct a biomolecular motor able to sense the Tau presence.

Additionally, rational design and assembling of peptides enabled their usage as modules to control the activity of viral proteases and modulate downstream signals, as demonstrated by Stein and Alexandrov (2014). Here, a modular design strategy was employed to develop a transducer protease connected to an auto-inhibitory (AI) peptide and containing different functional units able to modulate the activity of the auto-inhibited enzyme [41]. An AI domain was also used to create an allosterically regulated receptor protease based on an artificial peptide receptor (affinity clamp). Other studies demonstrated the suitability of viral protease-modules to create sensing and amplification circuits [42] and perform logic operations, like binary Boolean logic [43].

Besides rational design, de novo proteins and peptides proved the possibility to generate an unlimited number of orthogonal and composable modules. α-helical coiled coils (CCs) are probably the most used scaffolds for engineering synthetic PPIs, as the H-bonding patterning can be designed in a predictable fashion. Noteworthy, freely-available toolboxes, like Pcomp and SYNZIP, provide several validated CC pairs which can be used to compose PPI networks [44,45]. Previously, viral protease circuits were presented as a system limited to in vitro applications. The de novo design of peptides and proteins allowed the validation in vivo of these systems. A notable example is the split-protease-cleavable-orthogonal-CC-based (SPOC) system (Figure 1E), where the employment of de novo designed coiled coil (CC) peptides enabled the generation of modular signaling cascades based on proteolysis. Here, split proteases reconstitution is achieved thanks to the cleavage of linkers, placed among the target CC, enabling the realization of Boolean logic functions and construction of signaling pathways in mammalian cells [46].

Another system named CIPHR (cooperatively inducible protein heterodimer) used de novo design of heterodimers to regulate the association among proteins of choice, as split enzymes and transcription machines, allowing the execution of logic functions in vitro and in vivo (Figure 1C) [47]. Peptides were also designed de novo to develop molecular switches like LOCKR (latching orthogonal cage/key proteins) [48], a CC-based ‘cage’ that can interact either intra-molecularly with a ‘latch’ or inter-molecularly with a peptide ‘key’ (Figure 1B). After the ‘key’ displaces the ‘latch’ from the ‘cage’, functional motifs on the ‘latch’ could engage the interaction with the target. The same technology was used by Kirkpatrick and co-workers (2020) to co-localize LOCKR (co-LOCKR) in target regions in the genome exploiting two Cas9 complexes which are fused to the ‘key’ and to the ‘cage’- ‘latch’, respectively, and used it to specifically promote the transcription of target genes when the two moduli are bound to the DNA target site [49]. Other examples demonstrated the exploitability of coiled coils design to accomplish transcriptional control [31,50]. De novo designed coiled coil peptides were also used to produce a supramolecular scaffold enabling the generation of intracellular filaments able to encompass the *E. coli* cytoplasm. Pyruvate decarboxylase and alcohol dehydrogenase were targeted to this cyto-scaffold leading to enhanced ethanol production thanks to enzyme co-location (Figure 1H) [51].

## 4. Peptides as Building Blocks for Targeted Proteolysis

Peptides were already shown to be highly selective in the engagement of interactions with endogenous components. This ability was exploited to trigger protein degradation through the proteolysis-targeting chimera (PROTAC) technology, where a small molecule can be isolated and used to target endogenous proteins for degradation, through the linkage with different E3 ubiquitin ligase (Figure 1D) [52].

Protac-1 was composed by a β-TRCP E3-recruiting peptide linked to ovalicin, which targets the MetAP-2 enzyme, causing its ubiquitination and degradation in vitro [53]. Successively, peptidic PROTACs [54] and a poly-D-arginine sequence were used to induce protein degradation in vivo [55]. These first examples of PROTAC molecules played a pioneering role in establishing the technology and demonstrating that E3 ubiquitin ligase can be specifically targeted. Recently, a peptide-enabled PROTAC was shown to direct Kelch-like ECH-associated protein-1 (Keap1)-dependent degradation of Tau by the proteasome [56].

Due to the inaccessibility of some extracellular targets, lysosomal-targeting chimeras (LYTACs) were also developed (Figure 1G). Signal peptides can force the endosomal-lysosomal internalization of peptide-extracellular POI chimeras, triggering the degradation of the extracellular target protein [57]. Through the conjugation of a synthetic oligopeptide ligand, mannose-6-phosphonate (M6Pn), to serine or lysine residues on antibodies, authors successfully induced internalization and degradation of therapeutically relevant proteins. This technology is amenable to contrast extracellular protein deposition (aggregates)-related diseases; for instance, it has been successfully employed to degrade apolipoprotein E4 (ApoE4), the main cholesterol carrier recognized as the major genetic risk factor in Alzheimer’s disease [57].

Additionally, peptide-mediated protein degradation was used in hydrophobic protein tagging (HyT). Hydrophobic groups are guided and attached to a POI by POI-interacting peptides (to mimic a partially denatured protein), to induce permanent chaperone binding, and to exploit natural surveillance mechanisms which degrades misfolded proteins. In fact, some chaperones (Hsp70 and Hsp90) could recruit the co-chaperones E3 ligase CHIP which mediates protein ubiquitination and degradation [58,59]. This technique was also successfully used to induce degradation of the Tau protein in Alzheimer disease mouse models [60].

## 5. Combinatorial Libraries as Platforms for Peptides Isolation

Combinatorial libraries have been developed to identify sequences in short times, which can be further screened using different assays [61]. Peptides can be isolated from combinatorial libraries for their ability to behave as PPI mediators, POI interactors and/or enzyme inhibitors, using either in vitro or in vivo techniques.

Combinatorial libraries can be also used to identify synthetic biology scaffolds and to produce cell-cell adhesion toolkits and linkers [62,63]. Cell-cell adhesion can be engineered to produce multicellular systems and to design multi-component metabolic pathways and materials but requires a fine control over morphologies and patterns of adhering cells. Glass and co-workers (2018) generated a genetically encoded synthetic platform enabling multicellular self-assembly control in *E. coli* through cell-cell adhesion. The library relies on peptide nanobodies and antigens displayed on the outer bacterial membrane (Figure 1J). The system was orthogonal, composable and controllable enabling the construction of, as proved by the authors, “well-defined morphologies and patterns through homophilic and heterophilic interactions, lattice-like self-assembly, phase separation, differential adhesion, and sequential layering” [62].

As described earlier for split proteases, peptide linkers affect the structure and the functional properties (folding, proteolytic stability, flexibility and relative 3D orientation of individual domains) of chimera proteins. Grawe and collaborators (2020) developed a new DNA assembly strategy enabling the efficient fusion of protein domains with combinatorial linker sequences. This DNA assembly system, named iterative functional linker cloning (iFlinkC), relies just on the combined action of restriction enzymes and T4 DNA ligase and enable the sequential assembly of fusion proteins. iFLinkC functionality was demonstrated through the construction of synthetic protease switches. Linker space was highly dynamic as the induction of protease activity can vary by several folds depending on the linker sequence and lenght [63]. iFLinkC is envisioned to be applicable for the generation of any type of chimeric protein, enabling the identification of the optimal linker for maximizing switching behaviors, as required for the construction of efficient synthetic protease switches.

## 6. Perspectives on the Application of PPI-Based Synthetic Protein Circuits

The field of protein circuits is quite vast and comprises several approaches for the isolation and characterization of protein scaffolds suitable for synthetic circuit design, reflecting great interest in the development of this area of synthetic biology.

Synthetic proteins/peptides scaffolds are convenient for protein circuit design for several reasons: (i) the combination of 20 different amino acids in protein or peptide folds allows a high number of possible combinations in terms of interactions, which are also expanded by the use of non-canonical amino acid or post-translational modifications [35]; (ii) the versatility and dynamicity of the responses are higher compared to systems based on transcriptional outputs; (iii) the increasing knowledge on structural data, molecular dynamics simulation and protein structure prediction facilitate the construction of such circuits [64,65,66].

Importantly, protein circuits can be based solely on components which are constitutively expressed, and interact with each other only under desired circumstances to produce a fast cell response, without the need for downstream transcriptional activation, even if this area of research is still at the beginning.

Synthetic proteins and peptides are also remarkable candidates for developing new drugs, as they can be used to deliver functional domains to a POI as seen for PROTACs. As mRNA-based drugs seem to be riding the wave as human therapeutics, many companies and research laboratories are clearly investing in protein-based therapies, as highlighted in the THPdb (http://crdd.osdd.net/raghava/thpdb/; access date 25 October 2021) database curated by the Food and Drug Administration (FDA) [67]. This should be also accompanied by new technologies enabling a reduction of the costs for protein and peptide production on large scales, and the development of automated platforms for high-throughput testing with the support of bio-foundries.
